# First person – Adam Farmer

**DOI:** 10.1242/bio.061961

**Published:** 2025-04-02

## Abstract

First Person is a series of interviews with the first authors of a selection of papers published in Biology Open, helping researchers promote themselves alongside their papers. Adam Farmer is first author on ‘ *trithorax* is an essential regulator of cardiac *Hox* gene expression and anterior-posterior patterning of the *Drosophila* embryonic heart tube’, published in BiO. Adam conducted the research described in this article while a graduate research assistant (PhD student) in Dr Kristopher Schwab's lab at Indiana State University, USA. He is now a medical student in the lab of Dr Fen Lei Chang at Indiana University School of Medicine, investigating basic and translational research exploring underlying mechanisms in development and disease.


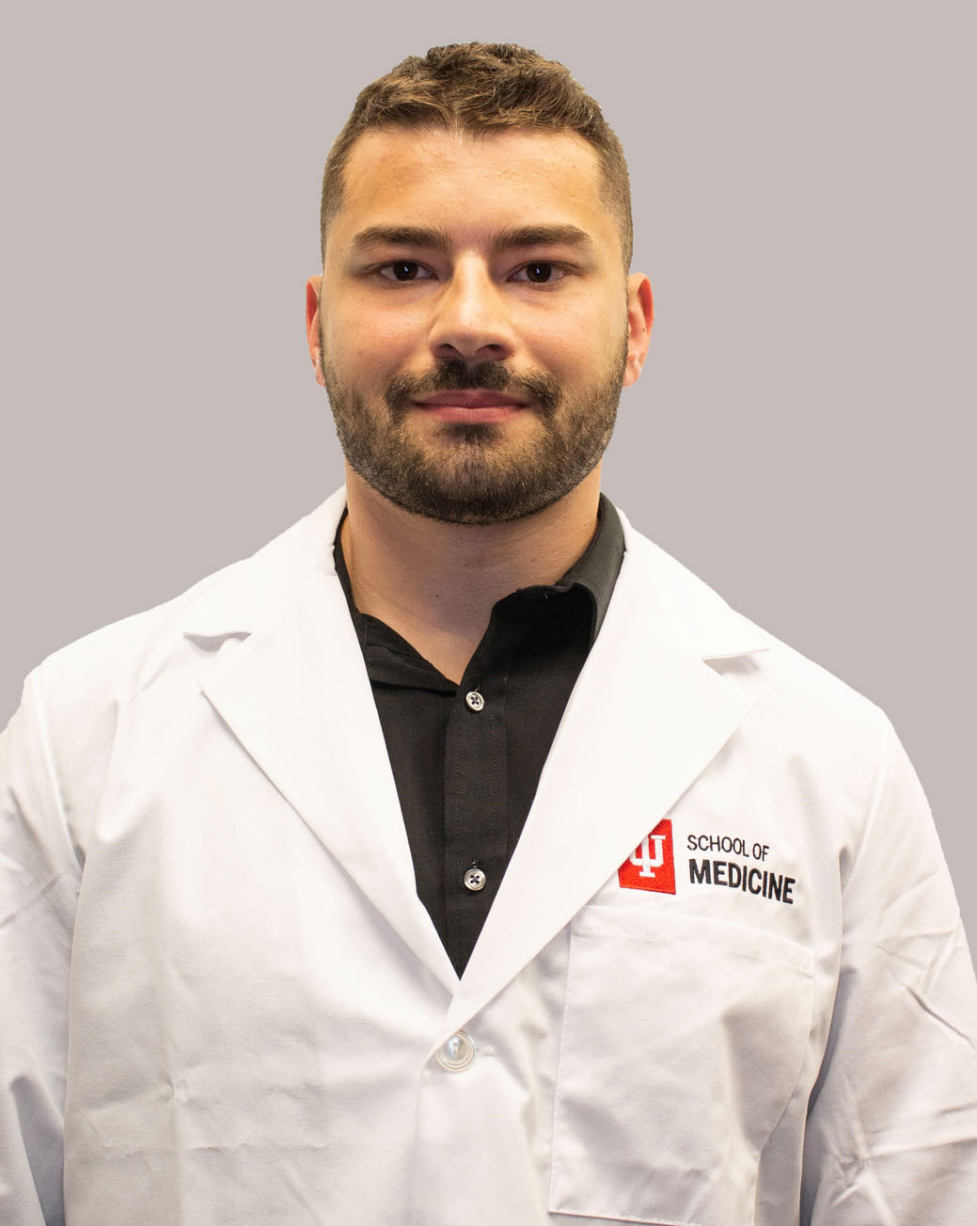


**Adam Farmer**

**Describe your scientific journey and your current research focus**

My scientific journey working on research began in undergrad at Purdue University where I worked in multiple nutrition labs. While I enjoyed the research process, it became clear with time that my true passion is understanding the underlying pathophysiological mechanisms involved in disease including those of developmental origin. As a result, I joined the lab of Dr Kristopher Schwab at Indiana State University to study cardiac development. During my time at Indiana State, I also had the chance to explore my interests in cancer research as well as being a Porter Cancer Research Center Fellow for 2 years.

My current research focus – that I began working on last summer as an IU School of Medicine SERF research program student under the guidance of Dr Fen Lei Chang – is the effectiveness of treating essential tremor in older adults (≥70 years of age) using MR-guided high-intensity focused ultrasound. Engaging in the project has increased my understanding of the translational aspects of research and its role in bridging the gap between laboratory findings and clinical applications in patients.

**Who or what inspired you to become a scientist?**

I began being interested in the human body at a young age. However, it was during my undergraduate years that my interests became more refined and developed into undercovering mechanisms involved in human disease. This came from a culmination of research experiences, classes, mentorship, and clinical. As a result, I was inspired to pursue a path that incorporated both the clinical and research components of medicine.

**How would you explain the main finding of your paper?**

Proper development requires the precise regulation of gene expression both at the right time and in the right place. Large protein complexes are essential for regulating gene expression during embryonic development, including in the heart. Our focus was on the essential founding members of the highly evolutionarily conserved COMPASS and COMPASS-like complexes. We found that the three key founding members of this family of protein complexes are essential for proper cell division in the fruit fly (*Drosophila melanogaster*) heart. One of these members, known as trithorax, was found to be critical for patterning the heart as well. The loss of trithorax lead to a dramatic transformation of one region of the heart into another. Overall, these findings suggest possible mechanisms that have yet to be investigated by which the human versions of trithorax may facilitate proper heart development and the errors that may occur in congenital heart disease.

**What are the potential implications of this finding for your field of research?**

Our findings demonstrate vital roles for the evolutionarily conserved COMPASS and COMPASS-like complexes in the regulation of heart development in fruit flies. Most importantly, our findings show that trithorax is a critical master regulator of cardiac patterning through the regulation of *Hox* activity and these findings improve our understanding of the genetic mechanisms by which *trithorax* orthologs control cardiac target gene expression in humans.

**Figure:**
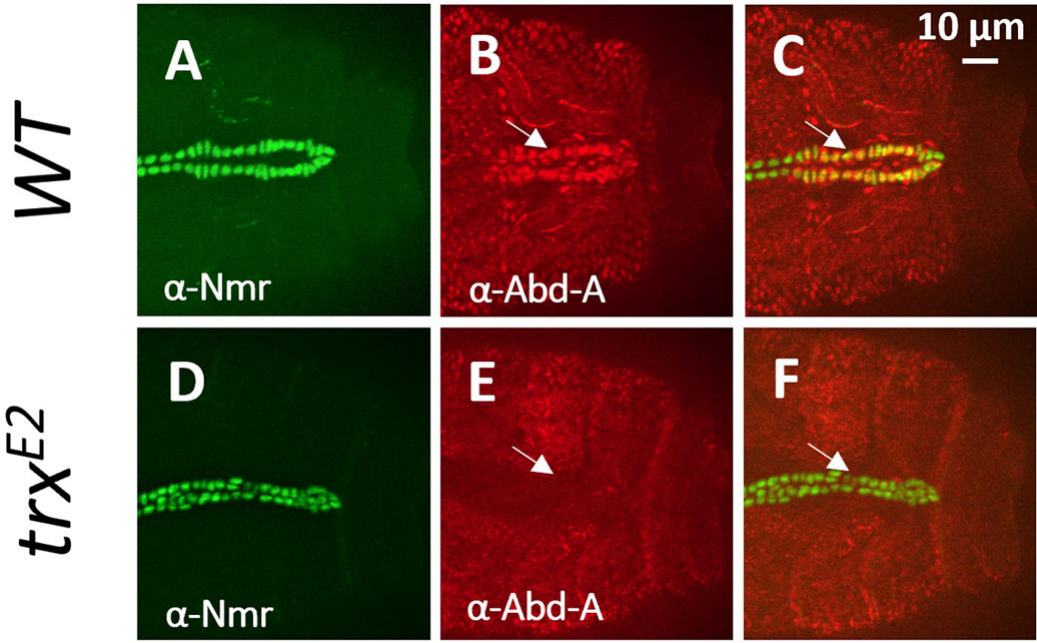
**(A-C) In the wild type, high Abd-A expression in the posterior dorsal vessel facilitates heart-proper patterning.** (D-F) In the *trx^E2^* null, Abd-A activity is completely absent within the posterior heart tube resulting with a striking homeotic transformation of the heart-proper into the anterior aorta.

**Which part of this research project was the most rewarding?**

The most rewarding aspect of this research project by far was finding the dramatic cardiac homeotic transformation within the *trithorax* mutant heart in my routine screen of *Drosophila* COMPASS and COMPASS-like mutants. Additionally, it was incredibly satisfying to see that our general knowledge of *Hox* regulation and activity aligns with the observed cardiac phenotypic transformations findings in our manuscript.

Additionally, presenting this research at various conferences through both oral and poster presentations was very fulfilling. Engaging with experts in the field at these conferences offered invaluable experiences as well and helped refine my understanding of our research and its broader implications.

**What do you enjoy most about being an early-career researcher?**

The most enjoyable aspect of being an early-career researcher is the broad range of options available for areas of research that I could further explore in both basic science and translational medicine without feeling confined to a specific area. I also find a great deal of fulfilment in building a research career that has the potential to contribute to a greater understanding of the underlying mechanisms in human disease and discoveries that can improve clinical care.

…focus on the aspects of research you can control while maintaining an appreciation and understanding of the things you can't

**What piece of advice would you give to the next generation of researchers?**

The most important piece of advice I would give to anyone pursuing research is to focus on the aspects of research you can control while maintaining an appreciation and understanding of the things you can't.

**What's next for you?**

I'm currently in medical school between my second and third year at IU School of Medicine. My main goal is to become a physician scientist and apply scientific exploration to advance clinical medicine. This dual role is appealing as it allows valuable opportunities to make an immediate difference in the lives of patients and advance clinical care through scientific discovery.
